# Management of Myeloproliferative Neoplasms: An Integrative Approach

**DOI:** 10.3390/jcm14145080

**Published:** 2025-07-17

**Authors:** Francesca Andreazzoli, Ilana Levy Yurkovski, Krisstina Gowin, Massimo Bonucci

**Affiliations:** 1 Hematology Department, North West Tuscany Hospital, 55041 Lucca, Italy; 2 ARTOI Foundation, Via Ludovico Micara, 73, 00165 Rome, Italy; maxbonucci@artoi.it; 3 Hematology Unit, Bnai Zion Medical Center, Haifa 3339419, Israel; ilana.e.levy@gmail.com; 4 Rappaport Faculty of Medicine, Technion—Israel Institute of Technology, Haifa 3200003, Israel; 5 Departments of Supportive Care and Hematology Hematopoietic Transplant, City of Hope Comprehensive Cancer Center, Irvine, CA 91010, USA; kgowin@coh.org

**Keywords:** myeloproliferative neoplasms, integrative hematology, acupuncture, mind–body therapies, microbiota

## Abstract

Myeloproliferative neoplasms (MPNs) are chronic blood cancers characterized by overproduction of blood cells, leading to increased thrombotic and ischemic risk. Patients frequently experience symptoms including fatigue, abdominal discomfort, and complications from thrombotic events, which significantly impact the quality of life (QoL). Many patients inquire about complementary and integrative medicine (CIM) approaches, including nutritional interventions and supplements, creating opportunities for healthcare providers to engage in meaningful discussions guided by the principle of safety. This review examines the current evidence for integrative approaches in MPN management, focusing on nutrition, microbiota, supplements, mind–body techniques, and acupuncture. We analyze the available data on anti-inflammatory interventions, QoL improvement strategies, and treatment tolerance enhancement. The review provides clinicians with evidence-based guidance for safely integrating complementary therapeutic approaches with conventional MPN treatment. This integrative approach represents an opportunity to develop more comprehensive and personalized therapeutic paradigms in hematology while ensuring that complementary interventions serve as adjuncts to evidence-based medical treatment.

## 1. Introduction

Myeloproliferative neoplasms (MPNs), including polycythemia vera (PV), essential thrombocythemia (ET), and primary myelofibrosis (PMF), are clonal hematopoietic stem cell disorders characterized by dysregulated hematopoiesis, chronic inflammation, and a variable risk of progression to acute leukemia [1,2]. Although therapeutic advancements have led to improved disease control and extended survival in some cases, MPNs remain incurable for most patients [3].

A central challenge in the management of MPNs is the high burden of physical and psychological symptoms, such as fatigue, pruritus, sleep disturbance, early satiety, mood disorders, and cognitive impairment. These symptoms are prevalent across disease subtypes and stages, often persist despite pharmacologic therapy, and significantly impair patients’ quality of life [4,5,6]. Conventional treatments, particularly the use of JAK inhibitors, while essential, frequently offer incomplete relief, and their side effects may further contribute to symptom distress [7].

In response, many patients turn to CIM to manage symptoms, enhance resilience—defined as the ability to adapt to and cope with the chronic and unpredictable nature of myeloproliferative neoplasms and their associated psychosocial stressors—and regain a sense of control. While resilience is frequently measured in cancer populations using general validated tools, such as the Connor–Davidson Resilience Scale [8], to our knowledge, no disease-specific definition or measurement of resilience currently exists for patients with MPNs. In the Survey of Integrative Medicine in Myeloproliferative Neoplasms (SIMM) study, over 50% of MPN patients reported using at least one CIM modality—most commonly aerobic activity, yoga, meditation, and acupuncture [9]. However, CIM use is often undisclosed to treating physicians, raising concerns about fragmented care, safety, and a lack of evidence-based integration into clinical practice [10].

Integrative oncology (IO) and integrative hematology (IH) are evolving frameworks that combine CIM with conventional care to address unmet supportive needs [11,12]. When delivered within a coordinated and scientifically informed framework, this approach has the potential to address the multifaceted symptom burden of MPNs and to support holistic patient care [13]. This evolving field, encompassing mind–body practices, lifestyle modification, nutritional strategies, and supportive interventions, warrants deeper exploration in the context of hematologic malignancies [14].

**Figure 1:**
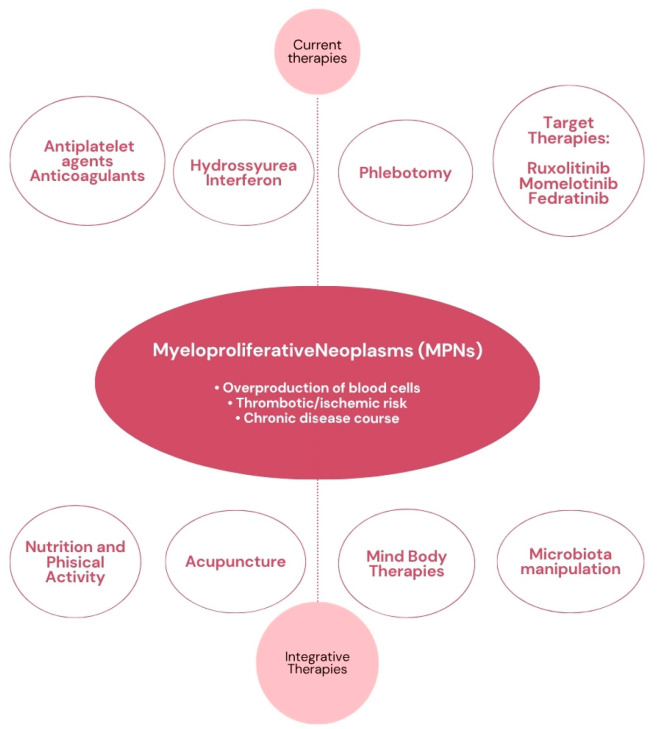
A potential integrative approach in MPNs.

This narrative review aims to synthesize the current evidence surrounding integrative medicine in MPNs. Unfortunately, as often occurs in this research field, the data we have available are often preclinical, and very few clinical studies exist, with randomized trials being completely lacking. Although this may be frustrating, particularly considering the great demand from patients for an integrative approach, the collected data serve primarily as a starting point for building recommendations that will need to be expanded with further clinical studies. Another aspect to consider is that, especially in light of new targeted therapies and possible drug interactions, it is very important to understand which substances may have a rationale and which could instead be potentially harmful to the patient. Exploring these aspects, together with the link to nutrition, lifestyle, and mind–body techniques, is the purpose of this review (Figure 1).

## 2. Nutrition, Microbiota, and MPNs

While the relationship between food and nutrition is increasingly evident for solid tumors [15], evidence on the impact of nutrition in hematology is lacking.

Research is early but evolving in hematologic disorders; for example, an interesting link between multiple myeloma (MM) and nutrition is emerging. While data are preliminary, a Mediterranean-style diet with a drastic reduction in animal and processed products is suggested to reduce the evolution from monoclonal gammopathy of undetermined significance (MGUS) to MM [16].

In MPNs, the data are limited regarding the impact of nutrition on outcomes. Nutrition interventions are particularly interesting in this population due to the chronicity of the disease and the possibility for cardiovascular event risk reduction. Patients who adopt a healthy eating pattern may modulate cardiovascular risk and potentially even reduce inflammation and consequent fibrosis. Moreover, patients themselves often ask the hematologist what they should eat, wanting to take partial active control over their own health, and if the hematologist provides comprehensive answers, patients will be more adherent to these recommendations [17].

### 2.1. Inflammation

Inflammation, in fact, has been recognized in MPNs as a critical element of disease pathogenesis, with increased inflammatory cytokines being a hallmark of the disease and contributing to symptomatology and disease progression [18,19]; the cytokine profile differs in the different disease subtypes, demonstrating the role they play in guiding toward a specific phenotype. The main cytokines involved appear to be IL-1β, IL6, IL8, VEGF, and TGFβ. Among these, TGFβ in particular is involved in the induction of bone marrow fibrosis. The JAK2 (V617F) mutation itself, pathognomonic of MPNs, is associated with constitutive activation of the NLRP3 inflammasome, leading to a chronic inflammatory state. Finally, inflammasome activation induces the same hematopoietic stem and progenitor cells mutated in MPNs to produce greater amounts of IL-1β, contributing to a pro-inflammatory bone marrow microenvironment. This chronic inflammatory state favors clonal expansion of mutated cells, creating a vicious cycle that perpetuates the disease [20]. Finally, age and aging processes are considered pro-inflammatory states accompanied by high levels of pro-inflammatory cytokines; this could contribute to the pathogenesis of MPNs, whose incidence increases with age [21].

Targeting inflammation-related pathways is the effect of many targeted therapies, starting from JAK inhibitors to the more recent BET inhibitors [22]. Since many patients cannot benefit from pharmacological therapy because, for example, they do not meet the criteria to start it or due to intolerance, other approaches aimed at reducing the inflammatory burden could potentially reduce the symptom burden and also slow the course of the disease by extinguishing its trigger.

Unlike the common Western diet, whose inflammatory impact is documented, a Mediterranean diet has the ability to reduce inflammation [23] but also improve the cardiovascular profile of patients whose thrombotic risk is already intrinsic to the disease [24]. Moreover, a diet low in animal proteins and rich in vegetables, legumes, nuts, whole grains, and oily fish has a favorable impact on the hematological patient through mechanisms other than anti-inflammatory ones, such as the reduction in IGF1, the reduction in glucose intake, and the anti-tumor properties of phytochemicals contained in the food itself [25].

### 2.2. IGF1 and Insulin

The role of IGF1 in MPNS has, to date, been little studied, but a connection has nevertheless been established. This growth factor is involved both in normal physiological erythropoiesis as well as in erythropoiesis caused by excessive activation of the JAK2 protein; inhibition of IGF1 receptors prevents Epo-hypersensitive erythroid cell colony formation in preclinical models [26]. The cross-link between JAK2 and IGF1 is well known; IGF1 promotes cell growth by activating the PI3K/AKT and MAPK pathways that overlap with those of JAK/STAT, amplifying them. Moreover, IGF1 can directly activate JAK2, and JAK2 hyperexpression seems to be linked to the greater expression of IGF1 receptors by PV progenitors [27]. IGF1 levels could potentially be monitored in patients with myeloproliferative neoplasms as a biomarker for nutritional intervention efficacy, particularly given that recent meta-analytic evidence demonstrates an association between IGF1 level ranges and all-cause mortality, with a specific 120–160 ng/mL range being associated with the lowest mortality. Serum IGF1 concentrations are modifiable through dietary changes, particularly through reducing animal protein and sugar intake while adopting plant-based dietary patterns [28]. The identification of this optimal IGF1 range provides a concrete therapeutic target for interventions; however, the clinical utility and cost effectiveness of serial IGF1 measurements in this specific patient population require further investigation to determine whether achieving and maintaining IGF1 levels within the 120–160 ng/mL range translates into improved quality-of-life outcomes and survival benefits in myeloproliferative neoplasm patients. Additionally, the potential use of IGF1-lowering agents, such as metformin, warrants evaluation in future studies, contingent upon establishing the clinical relevance of IGF1 modulation within this target range for MPNs management [29,30]. Pathological cells in MPNs, characterized by JAK2 mutation, exhibit enhanced glucose uptake and increased glycolytic activity compared to normal hematopoietic cells [31,32].

Furthermore, the JAK2/STAT5 pathway, once constitutionally activated, increases the expression of the inducible rate-limiting enzyme 6-phosphofructo-2-kinase/fructose-2,6-bisphosphatase 3 (PFKFB3), allowing greater use of circulating glucose, useful for its metabolism and survival [33]. This could support the rationale for a low-glycemic-load diet in these patients.

The Nutrient Trial has demonstrated the feasibility and adherence to a Mediterranean diet by patients with MPNs, with 80% of patients being able to maintain good adherence to the proposed dietary pattern. The authors also showed that, in the group following a Mediterranean diet compared to the one following the standard US Dietary Guidelines for Americans (USDA), there was an improvement in symptoms after only 10 weeks of intervention. The lack of changes in inflammatory cytokine levels was attributed to the small sample size and short intervention period [34].

### 2.3. Obesity

Obesity is involved in the pathogenesis of MPNs through many mechanisms. First of all, some evidence shows that it represents a risk factor for MPNs, particularly in patients with ET [35,36]. Obesity also contributes to the production of inflammatory molecules such as interleukin-6 (IL6) and tumor necrosis factor-α (TNFα), favoring the creation of an environment useful for MPNs’ progression [37]. The evidence that MPNs, even in the absence of obesity, generate a specific inflammatory cytokine signature, combined with the known inflammatory effects of obesity, suggests a potential for synergistic inflammatory effects when both conditions coexist [38,39]. However, the relative contributions of hematologic disease versus adiposity to symptom burden, particularly fatigue, remain to be elucidated through studies with appropriate BMI-stratified analyses.

While fatigue is reported by almost 90% of obese patients with MPNs [39], the lack of comparative data from non-obese MPN cohorts limits our ability to determine whether this high prevalence is primarily attributable to the underlying neoplasm, obesity-related factors, or their interaction; the cytokine most responsible for fatigue would be TNFα, which, in fact, is increased both in patients with MPN and in the general obese population [40,41]. The altered production of leptin and adiponectin could compromise the bone marrow microenvironment and also normal hematopoiesis [42]. Obese patients also present an altered insulin and metabolic pattern characterized by insulin resistance and increased IGF1 [43]; the latter in particular could contribute to the triggering or progression of altered molecular signals, as explained above, responsible for disease progression [28].

Finally, the obese phenotype increases cardiovascular risk through many pathophysiological mechanisms [44], and this adds to the risk already intrinsic to MPNs.

These data suggest that the Body Mass Index (BMI) is a modifiable factor to be taken into consideration in the treatment of MPNs that could impact not only the quality of life but also the outcome. At the same time, it should be noted that ruxolitinib, a drug widely used in MPNs, has been associated with weight gain in patients due to its interactions with leptin [45]. These data should be taken into consideration, and obese patients might need their medication dosage adjusted or could benefit from more individualized nutrition advice. At the moment, the implications are not entirely clear, and ad hoc prospective studies are deemed necessary to understand the metabolic implications of ruxolitinib therapy and also the correct management of this patient setting.

### 2.4. Malnutrition

On the other hand, malnutrition, which is frequent particularly among patients with PMF, can have many implications.

The relationship between BMI and symptom burden in MPN patients is complex and requires careful interpretation. While patients with normal BMI generally experience the best quality of life, those with low BMI show increased constitutional symptoms [5].

Importantly, this relationship likely reflects involuntary weight loss associated with disease progression, cachexia, or malnutrition rather than intentional weight management. In patients with PV, progressive involuntary weight reduction (defined as a decrease of more than 10% in BMI) has been associated with worse overall survival [46]; cachexia in general is associated with disease progression and can be one of its first signals [47]. This finding does not contradict the potential benefits of targeted nutritional interventions aimed at metabolic optimization, as the mechanisms and clinical contexts differ substantially. Involuntary weight loss in MPN patients typically reflects disease-related catabolism and poor nutritional status, whereas structured dietary approaches focus on metabolic pathway modulation while maintaining adequate nutritional support and lean body mass.

Malnutrition also impacts the outcome due to its effect of compromising the immune function, thus increasing the risk of infections but also of treatment tolerance.

Although the pathogenetic mechanism may differ partly from that present in obese patients, inflammation also plays an important role in this case [48]. Many scores have been attempted to test the degree of malnutrition, among which many included serum albumin concentration and lymphocyte count, as in the Prognostic Nutritional Index (PNI). Lucijanic et al. have demonstrated that PMF patients with low serum albumin levels exhibit several concerning clinical features: increased fibrosis severity, markers of more aggressive disease (elevated LDH, peripheral blood blasts, and transfusion dependence), higher inflammatory markers like C-reactive protein (CRP), and ultimately, shorter survival times [49]. The improvement in body weight and albumin secondary to therapy with ruxolitinib could partly contribute to the survival advantage in patients treated with the JAK2 inhibitor; the proposed mechanism for this off-target effect of ruxolitinib is a reduction in JAK2-mediated phosphorylation of STAT3 in the arcuate nucleus of the murine hypothalamus in response to feeding or exogenous leptin [50].

According to Lucijanic M et al., lymphocyte counts are typically reduced in primary myelofibrosis patients, and incorporating these parameters into the PNI score creates an independent survival predictor relative to the DIPSS classification, particularly for those with the most diminished PNI values.

Performing the measurement of these two parameters is feasible and economical, and this information could help identify which patients face the highest risk and would benefit most from nutritional support [49].

In conclusion, this U-shaped relationship between BMI and the course of MPNs highlights the importance of monitoring the nutritional status of the patient and potentially incorporating nutritional and lifestyle interventions alongside pharmacological treatment for MPNs.

### 2.5. The Role of Microbiota

Many of the effects of food pass through a remodeling of the microbiota, which acts as an interface between the host and the diet. In MPNs, we know that inflammation plays a fundamental role; it is possible to hypothesize that, being a close link between microbiota and inflammation, the microbiota could contribute to the inflammatory trigger typical of MPNs. On the other hand, inflammation itself can alter the microbiota in a dysbiotic direction.

Few data are known to date. Oliver et al. demonstrated that MPN patients have a distinct microbiota composition compared to healthy individuals, characterized by lower levels of Phascolarctobacterium bacteria and reduced concentrations of short-chain fatty acids, particularly propionate. The reduction in Phascolarctobacterium is also characteristic of autoimmune diseases, where it plays a role in modulating inflammation [51]. In contrast, a different study comparing microbiota samples from 25 MPN patients and 23 healthy controls found no significant differences between these groups and similarly detected no microbiota distinctions between patients with high versus low symptom burden. The only notable finding was a 20% higher abundance of bacteria from the Prevotellaceae family in MPN patients; notably, patients receiving ruxolitinib therapy showed distinct microbiome profiles compared to those treated with hydroxyurea [52]. Recent research has shown that PV patients exhibit decreased alpha diversity in their gut microbiome and lower proportions of various Firmicutes bacterial taxa compared to healthy individuals; interestingly, patients treated with interferon had a microbiota composition more similar to that of healthy subjects, suggesting what has already been published, namely that IFN, as an anti-inflammatory agent, may be able to restore intestinal permeability [53,54]. Intestinal permeability does indeed appear altered in patients with PV, as demonstrated by the increase in circulating lipopolysaccharide (LPS) compared to healthy subjects [55].

Overall, the studies we have available are few and have not clarified to date the role that microbiota may have in the pathogenesis and perpetuation of the disease, as demonstrated for other cancers. However, we believe that these data, although preliminary, suggest that the role of the microbiota in MPNs should not be overlooked.

In general, we can affirm, based on the data present in the literature, that inflammation plays a key role in the pathogenesis of the disease, and a dietary approach aimed at reducing inflammation, with a role partly mediated by the microbiota, may have a beneficial role. Also paying attention to the nutritional status of the patient, the possible cardiovascular risk added by being overweight and diet, as well as the effect of the drugs used, must be taken into consideration.

## 3. The Role of Supplements in MPNs

The use of supplements is increasingly widespread and recognized among patients with lymphoma and myeloma, while data for MPNs are emerging; the results from the Nutrient Survey show that 72% of MPN respondents reported the use of supplements [56]. The most commonly used supplements by patients according to this survey are amino acid supplements, N-acetyl cysteine (NAC), Bach flower remedies, vitamin D, multivitamins, omega-3 fatty acids, calcium, turmeric, green tea, vitamin E, medical marijuana, and medicinal mushrooms. Comparable results also emerge from the SIMM study [9]. In most cases, patients do not report their use to physicians or, in any case, are not advised by physicians on which supplements to use. On the other hand, healthcare professionals do not have access to much evidence-based data, as most studies, as we will see, are based on preclinical models (cell and mouse models). A review of potentially beneficial supplements or those potentially harmful—for example, due to drug interactions—is therefore the purpose of this section.

### 3.1. Curcumin

In recent years, curcumin, a component of Curcuma Longa root, has shown its pleiotropic activity due to its ability to modulate many intracellular signal pathways.

#### 3.1.1. Preclinical Studies

In MPNs, its action works through many mechanisms. The main one is the anti-inflammatory effect: as we have seen, inflammation plays a key role in the pathogenesis of MPNs, and curcumin has a well-demonstrated anti-inflammatory effect [57], contributing to reducing the cytokine burden. Curcumin can also induce apoptosis while simultaneously inhibiting anti-apoptotic proteins, such as Bcl2 and NFkB [58]. It has been demonstrated that in chronic myeloid leukemia, its anti-apoptotic action occurs through the upregulation of the PTEN oncogene, which is a target of miR-21, overexpressed in many cancer cells [59]. Its ability to also inhibit NFkB, a crucial factor in the activation of inflammatory, proliferative, and anti-apoptotic pathways, could play a key role [58]. Finally, it inhibits the JAK/STAT pathway, constitutively activated in MPNs [60,61].

#### 3.1.2. Clinical Evidence

Despite promising preclinical results, clinical studies are lacking and necessary to understand the potential benefit of this substance in MPN patients and the possible interactions with JAK inhibitors and new classes of drugs. While data on pharmacokinetics and safety are available, as well as new high-availability formulations, the possible interaction with anticoagulant or anti-platelet drugs (almost always used in MPN patients) represents a difficult limitation to overcome in this patient setting; large-scale, randomized clinical trials are therefore necessary to define whether and how to use this valuable supplement. The NCT06063486 trial is currently ongoing with the aim of evaluating changes in inflammatory response and symptomatology in patients with clonal cytopenia of undetermined significance (CCUS), low-risk myelodysplastic syndrome (LR-MDS), and MPNs [62].

### 3.2. Vitamin D

Vitamin D deficiency is very common in the population and is associated with a higher incidence of cancer; in MPNs, lower vitamin D levels occur particularly in patients with PMF, and severe deficiency (levels below 10 ng/mL) occurs in patients with PMF and ET. However, the deficiency does not seem to correlate with clinical or laboratory variables, nor with OS or leukemia-free survival [63].

#### 3.2.1. Preclinical Studies

Preclinical studies have provided conflicting evidence regarding vitamin D’s role in MPNs. There is limited evidence that vitamin D may interfere with the JAK2/STAT pathway [64]. However, murine models have demonstrated that vitamin D appears to stimulate bone marrow fibrosis through bone marrow macrophages, promoting the proliferation of collagen-producing fibroblasts and consequent reversible bone marrow fibrosis. Preclinical models lacking the vitamin D receptor, as well as vitamin D deprivation itself, are not associated with myelofibrosis, which would be independent of TGFβ1 or megakaryocytes [65]. These findings contradict previous observations regarding the presence of bone marrow fibrosis in children with rickets [66], which was reversible after vitamin D supplementation, as well as the proven ability of vitamin D to reduce circulating TGF β1 [67].

#### 3.2.2. Clinical Evidence

Clinical data on vitamin D in MPNs remain limited. Vitamin D could be beneficial due to its protective role against thrombosis and may therefore reduce cardiovascular risk in MPN patients [68]. Additionally, there is preliminary evidence that vitamin D can reduce platelet count in ET patients [69]. Although supplementing vitamin D in deficient patients might be recommendable, the evidence that vitamin D can stimulate bone marrow fibrosis makes the correct therapeutic approach controversial.

More data are needed to better understand vitamin D supplementation in MPNs, and an investigation of vitamin D receptor antagonists as anti-fibrotic agents could be considered.

### 3.3. Omega-3 Fatty Acids

#### 3.3.1. Preclinical Studies

Omega-3 fatty acids, particularly eicosapentaenoic acid (EPA) and docosahexaenoic acid (DHA), have well-established anti-inflammatory effects that contribute to reducing the cytokine burden and resolving inflammation through specialized pro-resolving mediators (SPMs) [70]. Due to their anti-platelet aggregation effect, they can attenuate the hypercoagulable state characteristic of MPNs [71]. Additionally, preclinical studies have demonstrated that omega-3 fatty acids are able to reduce myeloid progenitors and increase cell differentiation [72].

#### 3.3.2. Clinical Evidence

Although there are no specific clinical studies on omega-3 fatty acids in MPNs, recent clinical evidence has addressed the safety concerns regarding their use. Claims regarding a possible enhancement of anti-platelet action have recently been refuted by a large meta-analysis [73]. Therefore, the use of omega-3 fatty acids could be considered due to their established anti-inflammatory effect and safety profile.

### 3.4. N-Acetylcysteine (NAC)

NAC is a sulfhydryl-containing compound with notable antioxidant properties that demonstrates several mechanisms of potential benefit in MPNs.

#### 3.4.1. Preclinical Studies

It has a potent antioxidant effect as a precursor of glutathione, which is particularly relevant, since elevated reactive oxygen species (ROS) levels have been demonstrated in MPNs, amplified by JAK2 mutations [74]. NAC is also characterized by anti-inflammatory properties and inhibitory effects on NFkB [75,76]. Additionally, preclinical studies have shown anti-fibrotic action in other organs, such as liver and lung, although studies specifically on bone marrow are lacking [77].

#### 3.4.2. Clinical Evidence

Currently, there are limited clinical data specifically addressing NAC use in MPN patients. Orally soluble formulations with enhanced bioavailability and intravenous formulations are now available. While formal clinical trials in MPN patients are lacking, preliminary clinical observations suggest good tolerability, although systematic evaluation remains necessary.

### 3.5. Artemisinin

Artemisinin is a sesquiterpene lactone derived from the plant Artemisia annua, used for centuries in traditional Chinese medicine.

#### 3.5.1. Preclinical Studies

Preclinical evidence suggests that it may have specific anti-fibrotic action in addition to its anti-inflammatory and proposed anti-cancer properties. The potential mechanisms through which it would exert its action in MPNs include JAK2/STAT3 downregulation [78] and formation of iron-dependent free radicals: artemisinin can cause cell death through the generation of reactive oxygen species from iron, a molecule in which cancer cells are particularly rich, making them more susceptible to so-called ferroptosis [79].

A preclinical study on zebrafish demonstrated that artemisinin can block erythroid production in cells carrying the JAK2 AV581F mutation, the zebrafish counterpart of the JAK2 V617F mutation in human PV cells [80]. Anti-fibrotic action has been demonstrated on pulmonary, renal, and hepatic tissue and is mediated by inhibition of TGFβ, inflammation, epithelial–mesenchymal transition (EMT), and myofibroblast activation. Since the anti-fibrotic mechanism seems to be common to all tissues, this could also occur in the bone marrow, although specific studies still need to be conducted [81].

#### 3.5.2. Clinical Evidence

Artemisinin has demonstrated a favorable safety profile in malaria therapy. Although preliminary clinical observations in MPN patients suggest acceptable tolerability and potential stabilization of blood counts, these findings require validation through controlled clinical studies.

### 3.6. Vitamin C

In a real-world analysis, vitamin C deficiency was demonstrated in 17% of patients with myeloid hematological diseases; the deficiency was more frequent in young people, in association with the AXL1 mutation or with acute myeloid leukemia [82].

#### Clinical Evidence

Vitamin C appears to particularly have a role in myelodysplastic syndromes, acute myeloid leukemias, and clonal cytopenias due to its epigenetic action through the modulation of TET2 [83] and synergy with azacitidine or decitabine [84,85].

In myeloproliferative diseases, however, its role is controversial. TET2 mutations have also been found in ET, PV, and PMF, although their prognostic impact appears limited [86].

Since vitamin C could potentially increase iron absorption and exacerbate red blood cell production in PV, it is typically not recommended for PV patients.

The observation that intravenous vitamin C administered for many consecutive days causes an alteration in thromboelastogram and platelet function raises further caution in using vitamin C in ET patients as well [87].

Overall, despite the positive role in some myeloid neoplasms, its role in myeloproliferative diseases is debated, and without consistent data based on evidence at the moment, its use in these pathologies is not recommended but requires further study.

### 3.7. Quercetin

Quercetin is a flavonoid compound found in many vegetables, belonging to the class of polyphenols; it is studied for its potential health benefits, among which the main ones are anti-inflammatory and antioxidant.

#### 3.7.1. Preclinical Studies

Quercetin has been shown to synergize with BET inhibitors in vitro, which is particularly relevant, since MPNs recognize inflammation as a fundamental trigger, and BET pathway inhibitors are under investigation for approval [88].

Quercetin also has the ability to promote apoptosis and autophagy through the modulation of PI3K/Akt/mTOR, Wnt/β-catenin, and MAPK/ERK1/2 pathways [89] and can inhibit the JAK2 pathway [90]. Through anti-inflammatory action, inhibition of TGFβ, and modulation of myofibroblast formation, quercetin presents itself as an anti-fibrotic agent that could potentially be effective in myelofibrosis, although preclinical and clinical studies confirming this hypothesis are currently lacking [91].

#### 3.7.2. Clinical Evidence

A clinical study is currently underway that involves the association of dasatinib and quercetin in idiopathic pulmonary fibrosis, precisely by virtue of their senolytic and anti-fibrotic action [92].

Based on these mechanisms of action and considering the availability of liposomal formulations with greater bioavailability, quercetin appears as a potential therapeutic tool in MPN patients requiring further study.

## 4. Acupuncture in MPNs

Acupuncture has been explored as a supportive therapy in MPNs, with patient surveys indicating interest in its use for symptom relief. The SIMM Study-2, a survey-based study, highlighted that patients with MPN actively seek acupuncture treatments to complement conventional care and enhance their QoL [9]. While data on acupuncture in MPN remain limited, case reports and broader IO research support its potential benefits.

Pruritus, a debilitating symptom in PV, is often resistant to conventional treatment. Although not specifically studied in MPN patients, acupuncture has been shown to improve this sensation in various diseases via inhibition of peripheral and central transmission of itching [93]. A case report documented notable symptom relief in a patient with PV following acupuncture treatment, highlighting marked improvements in itching, fatigue, bone pain, and headache. These findings suggest a potential role of acupuncture in modulating neuroimmune responses and alleviating inflammation [94]. Similarly, erythromelalgia, a painful microvascular disorder seen in ET and PV, has shown responsiveness to acupuncture and Kampo medicine in a case report, supporting its vasoregulatory and analgesic effect [95]. Fatigue, another hallmark symptom of MPN, has been a focus of integrative interventions, including acupuncture, in both solid and hematologic malignancies, although not specifically studied in MPN patients [96,97]. The proposed mechanisms of action involve anti-inflammatory effect and neuromodulation [98]. Additionally, acupuncture has been studied in related hematologic malignancies and bone marrow transplanted patients, demonstrating its efficacy in improving nausea and vomiting, sleep disturbances, QoL, and overall well-being [98,99], reinforcing its potential in MPN management.

While acupuncture is generally safe, careful consideration is required in MPN patients, particularly those with thrombocytopenia or an elevated risk of bleeding. The risk of infection is low, but immunocompromised patients should receive treatment from trained professionals under strict aseptic conditions [100,101]. Evidence from hematologic malignancies suggests that acupuncture can be safely administered even in patients with low platelet counts (below 20 × 10^9^/L), reinforcing its feasibility in patients with MPN [102]. In conclusion, acupuncture presents a promising adjunctive therapy for MPN-related symptoms, addressing unmet needs in symptom management and patient well-being. Further clinical studies are warranted to establish standardized protocols and expand its evidence base in this patient population.

## 5. Physical Activity in MPNs

Most exercise-focused research in hematologic cancers has concentrated on leukemia, lymphoma, and multiple myeloma, though emerging data now point to benefits for MPN patients as well [103]. Physical activity has been shown to mitigate a range of cancer-related adverse effects, including fatigue, cognitive changes, psychosocial distress, sexual dysfunction, and overall declines in the quality of life [104]. Furthermore, individuals who maintain regular physical activity have a reduced risk of several malignancies, such as colorectal, liver, esophageal, lung, leukemia, and melanoma [105]. In addition, exercise has been associated with decreased cancer-specific and all-cause mortality, particularly in those with breast and colorectal cancer [106].

Mechanistically, exercise may enhance the immune function, potentially explaining its anti-cancer effects. For instance, the mobilization of natural killer (NK) cells and IL-6 release during physical activity has been linked to tumor suppression in preclinical models [107,108,109]. Moreover, exercise has been shown to modulate insulin-related growth factors that may play a role in cancer progression, especially in breast cancer [110]. Other theorized mechanisms include improved treatment tolerance leading to greater therapy completion, as well as beneficial changes in the epigenome [111,112].

In the context of MPNs, exercise may be especially important due to the heightened risk of thrombotic events, with cardiovascular fitness potentially improved through enhanced fibrinolysis [113]. Sedentary behavior in this population has been linked to worse fatigue and diminished quality of life [114,115]. A large, international, multicenter patient reported survey suggested that structured aerobic and resistance training was associated with reduced symptom burden (*p* = 0.01) and lower rates of depression (*p* = 0.006), though other studies have reported mixed results [116]. One 12-week exercise trial in MPNs found no significant improvements in fatigue or quality of life, possibly due to differences in adherence, patient characteristics, or trial design [113]. Nonetheless, improved physical strength (*p* = 0.01, *p* < 0.001) and increased VO_2_ max (*p* = 0.01) were observed, highlighting the feasibility and impact of exercise in this population [117].

The optimal type, frequency, and intensity of exercise for MPNs remains unclear. The current guidance often draws from NCCN recommendations for cancer survivors, which advocate for 150 min of moderate aerobic activity and strength training weekly, spread over at least two days [118]. Among polycythemia vera patients, preferences tend to lean toward individualized, outdoor-based programs performed twice weekly for 45–60 min [119]. Exercise plans may need adjustment for individuals with splenomegaly, bleeding risks, or pain, such as avoiding high-impact sports. Barriers to participation are common, and addressing these through cognitive support and personalized exercise plans may improve engagement [119,120].

## 6. Mind–Body Therapies in MPNs

Mind–body therapies (MBTs) integrate mindful awareness, physical movement, and breathing techniques to support psychological and physiological health [121]. Approaches such as yoga, mindfulness/meditation, tai chi, and qigong have demonstrated benefits in alleviating cancer-related symptoms like anxiety, depression, pain, and sleep disturbances [122]. These effects are believed to occur through multiple pathways, including dampening systemic inflammation, as evidenced by reductions in CRP and IL-6, and transcriptional shifts that downregulate NF-kB activity while enhancing glucocorticoid receptor signaling [123]. MBTs may also influence central and autonomic nervous system regulation, fostering self-compassion and reducing maladaptive thought patterns [124].

Yoga, for example, has been shown to improve psychosocial outcomes in patients with breast cancer and lymphoma [125]. In the MPN setting, a 12-week online yoga program (60 min per week) led to improvements in sleep, symptom burden, mood, and pain [126]. Patients reported benefits in circulation, respiratory function, dietary habits, and enjoyment [127,128]. These findings are supported by high levels of satisfaction, adherence, and follow-up [129]. Biologically, yoga reduced plasma TNF levels in MPNs, although IL-6 remained unchanged, with benefits more prominent in individuals with higher BMI [130]. Some participants experienced discomfort, such as spleen irritation, but these were manageable through modified poses [131]. Barriers to participation can often be mitigated by remote formats and low-dose interventions. To date, recommendations regarding yoga style, frequency, and intensity remain vague, though Hatha and Vinyasa practices of mild to moderate intensity are commonly used. Notably, sessions exceeding 60 min weekly did not yield additional benefits, highlighting the need for larger, controlled trials [130,131].

Mindfulness, defined as an intentional, non-judgmental awareness of the present moment, has also gained traction as an intervention in MPNs [132]. Mobile apps like Calm and My Wellness Coach (MWC) have demonstrated feasibility and patient satisfaction, with users often recommending these tools to peers [133,134]. Evidence suggests that mindfulness apps can reduce anxiety and depressive symptoms, particularly among patients with pre-existing mental health challenges [133,134]. MWC also appeared to improve symptoms such as dizziness, night sweats, sexual dysfunction, bone pain, cognitive difficulties, and sedentary behavior. Participants noted enhanced tranquility and overall well-being, though improvements in sleep were limited, perhaps due to shorter intervention durations (4–12 weeks) [133,134].

Technical limitations like poor internet access, annoying app notifications, and tech literacy challenges, especially in older populations, were noted [135,136]. Optimal dosage remains uncertain, as no significant difference in outcomes was observed between users engaging in 10 versus 30 min of mindfulness per session [137].

## 7. Other Integrative Approaches for MPNs

Integrative therapies, such as massage, music therapy, art therapy, Aryuveda, healing touch, and other therapies, are increasingly recognized for their potential to alleviate symptom burden and enhance QoL in patients with cancer. These integrative modalities may offer supportive benefit by reducing psychological sequelae of the MPNs while concurrently treating the physical contributors of symptom burden associated with MPN. Despite promising preliminary evidence in other cancer populations, there is a critical gap in rigorous research evaluating the efficacy, feasibility, and mechanistic impact of these therapies in MPNs. Expanding clinical trials and implementation studies in this area could inform integrative care models and help optimize patient-centered outcomes in the MPN population.

## 8. Discussion

In this review, we attempted to collect all the available literature data of scientific value on the various tools of integrative medicine and their potential role in MPNs. The current landscape of integrative medicine in MPN management is characterized by significant research gaps that must be systematically addressed to advance clinical practice. The predominance of preclinical investigations over clinical studies represents the most critical limitation in this field. This evidence deficit stems from the relatively recent recognition of integrative approaches in hematology and the inherent complexity of conducting controlled trials in rare disease populations.

The scarcity of disease-specific clinical evidence for MPN patients represents a fundamental challenge. While general principles from oncology and other chronic conditions may be applicable, the unique pathophysiology of MPNs—particularly their inflammatory nature and JAK/STAT pathway involvement—demands targeted research approaches. The current gaps include the lack of standardized protocols for dietary interventions, insufficient safety data for supplement use in the context of standard therapies, and limited understanding of optimal timing and dosing for integrative interventions.

Despite these limitations, integrative medicine offers significant potential for MPN management. The inflammatory nature of MPNs provides a strong rationale for anti-inflammatory dietary interventions and lifestyle modifications that may address both symptoms and disease progression. Moreover, integrative approaches represent a valuable opportunity for patients to actively participate in their care journey, potentially enhancing their sense of empowerment and overall well-being while addressing the multidimensional aspects of their condition. However, any recommendations in this domain must adhere to the fundamental principle of “Primum non nocere” (First do no harm), ensuring that integrative interventions serve as valuable adjuncts to established evidence-based treatments.

The current research priorities center on understanding the anti-inflammatory potential of dietary modifications, particularly the Mediterranean diet, given the established inflammatory trigger in MPNs. The investigation of specific compounds with JAK/STAT pathway modulatory effects—including artemisinin, curcumin, and NAC—represents another critical area. Additionally, symptom management through non-pharmacological approaches, such as acupuncture and mindfulness interventions, shows promise for improving quality of life outcomes.

The advancement of integrative medicine in MPN care requires collaborative efforts between hematologists, integrative medicine specialists, and researchers to establish a robust evidence base that ensures patient safety while maximizing therapeutic potential. This coordinated approach will enable the development of comprehensive treatment strategies that address both the biological and psychosocial aspects of MPN management. Table 1 summarizes the potential integrative approaches and research directions in the MPN setting.

## 9. Conclusions

In conclusion, integrative therapies hold significant promise for improving the symptom burden and quality of life among patients with MPNs. However, rigorous research is urgently needed to evaluate the effects of key integrative interventions, such as nutrition, physical activity, dietary supplements, and mind–body practices, on both subjective and objective outcomes. Beyond symptom control, future studies must examine how these interventions influence critical clinical endpoints, including thrombosis, leukemic transformation, and overall survival. To fully realize the potential of integrative care in MPNs, robust translational research is essential. Such efforts should investigate the biological underpinnings of integrative interventions, including their effects on mutational allele burdens, clonal evolution, cardiovascular risk markers, inflammatory pathways, immune phenotypes, and the microbiome. Through multidisciplinary collaboration and thoughtfully designed trials, we can bridge the gap between integrative approaches and precision medicine, advancing whole-person care for those living with MPNs.

## Figures and Tables

**Table 1:** Potential integrative approaches and research directions in MPN management.

Modality	Mechanism	Contraindications	Research Priorities
Disease Management			
Mediterranean diet	Anti-inflammatory, ↓CV risk [23,24]	Safe	RCT: inflammation markers, BMI, QOL [5,39]
Vitamin D	↓Thrombosis risk [68]; JAK/STAT interference [64]	BM fibrosis interaction [65,66,67]	Dosing studies, serum monitoring
Curcumin	Curcumin JAK/STAT inhibitor [57,58,59,60,61]	Anticoagulant interaction	Safety/efficacy trials, drug interactions
Omega-3	Anti-inflammatory [71]; ↓hypercoagulability [71]	Safe	Optimal dosing, bleeding risk assessment
NAC	Antioxidant [74]; ↓ROS, NFκB [75,76]	Safe	IV vs. oral trials, dose-response
Artemisinin	JAK/STAT inhibitor [78,79]	Low toxicity	Phase I/II trials in MPN
Quercetin	JAK2/BET modulator [84,85,86]	Unknown	Safety/efficacy trials, drug interactions
Vitamin C	Unclear in MPN	May worsen PV [87]	Safety/efficacy trials
**Symptom Management**			
Mindfulness	↑QOL, symptom relief [133,134]	Technical limitations [135,137]	RCT: standardized protocols, QOL
Yoga	↓TNF [130]	Spleen irritation [131]	Protocols for MPN
Physical Activity	↓Fatigue, ↑strength [116,117]	Safe	Exercise prescription trials
Acupuncture	Pruritus, fatigue, pain relief [93,94,95,96,97,98,99]	Bleeding/infection risk [100,101]	RCT: inflammation markers, QOL
Touch/Music/Art	Symptom relief, mood, QOL	Safe	Comparative effectiveness research

Abbreviations: BET = bromodomain and extra-terminal motif; CV = cardiovascular; JAK/STAT = Janus kinase/signal transducer and activator of transcription; NAC = N-acetylcysteine; PV = polycythemia vera; QOL = quality of life; ROS = reactive oxygen species; ↓ = reduced; ↑ = increased.

## Data Availability

This review article did not generate any new data.

## Abbreviations

The following abbreviations are used in this manuscript:

BET	Bromodomain and Extra-Terminal Motif
BCL2	B-Cell Lymphoma
BMI	Body Mass Index
CIM	Complementary and Integrative Medicine
CRP	C-Reactive Protein
DHA	Docosahexaenoic Acid
DIPSS	Dynamic International Prognostic Scoring System
EPA	Eicosapentaenoic Acid
Epo	Erythropoietin
ET	Essential Thrombocythemia
GLUT1	Glucose Transporter 1
IGF1	Insulin-Like Growth Factor 1
IH	Integrative Hematology
IL-1β	Interleukin-1β
IL6	Interleukin 6
IL8	Interleukin 8
IO	Integrative Oncology
JAK	Janus Chinasi
MAPK	Mitogen-Activated Protein Kinase
MGUS	Monoclonal Gammopathy of Undetermined Significance
MM	Multiple Myeloma
MPNs	Myeloproliferative Neoplasms
NAC	N-Acetyl Cysteine
OS	Overall Survival
PFKFB3	6-phosphofructo-2-kinase/fructose-2,6-bisphosphatase 3
PFS	Progression-Free Survival
PMF	Primary Myelofibrosis
PNI	Prognostic Nutritional Index
PTEN	Phosphatase and Tensin Homolog
PV	Policytemia Vera
QoL	Quality of Life
SIMM	Survey of Integrative Medicine in Myeloproliferative Neoplasms
STAT	Signal Transducer and Activator of Transcription
TET2	Tet Methylcytosine Dioxygenase 2
TGF	Transforming Growth Factor
TNF α	Tumor Necrosis Factor-α
USDA	US Dietary Guidelines for Americans
VEGF	Vascular Endothelium Growth Factor
